# Dimerization Promotes PKR Activation by Modulating Energetics of αC Helix Conversion between Active and Inactive Conformations

**DOI:** 10.1021/acs.jpcb.4c02460

**Published:** 2024-09-18

**Authors:** Aaron G. Feinstein, James L. Cole, Eric R. May

**Affiliations:** † Department of Molecular and Cell Biology, University of Connecticut, Storrs, Connecticut 06269, United States; ‡ Department of Chemistry, University of Connecticut, Storrs, Connecticut 06269, United States

## Abstract

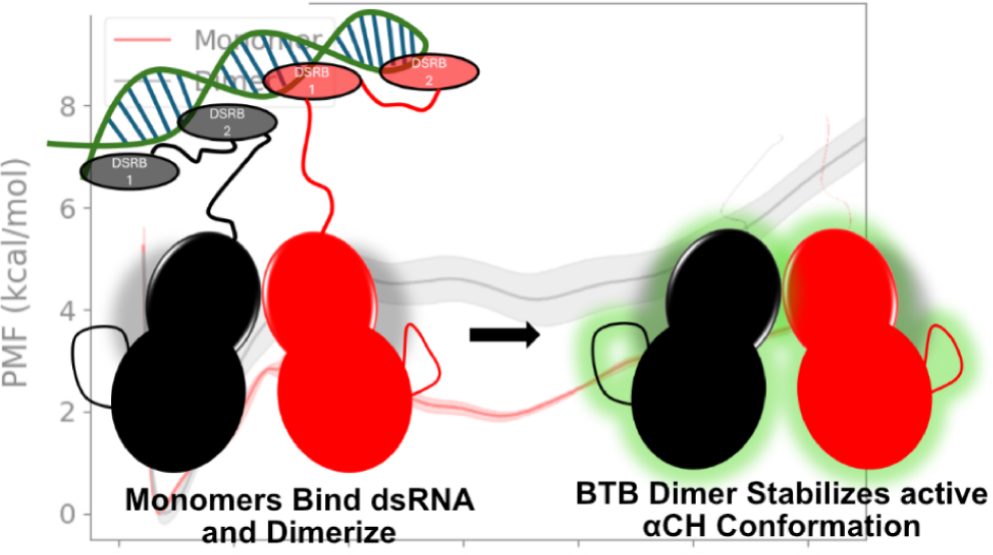

Protein kinase R (PKR) functions in the eukaryotic innate immune system as a first-line defense against viral infections. PKR binds viral dsRNA, leading to autophosphorylation and activation. In its active state, PKR can phosphorylate its primary substrate, eIF2α, which blocks the initiation of translation in the infected cell. It has been established that PKR activation occurs when the kinase domain dimerizes in a back-to-back configuration. However, the mechanism by which dimerization leads to enzymatic activation is not fully understood. Here, we investigate the structural mechanistic basis and energy landscape for PKR activation, with a focus on the αC helix—a kinase activation and signal integration hub—using all-atom equilibrium and enhanced sampling molecular dynamics simulations. By employing window-exchange umbrella sampling, we compute free-energy profiles of activation, which show that back-to-back dimerization stabilizes a catalytically competent conformation of PKR. Key hydrophobic residues in the homodimer interface contribute to stabilization of the αC helix in an active conformation and the position of its critical glutamate residue. Using linear mutual information analysis, we analyze allosteric communication connecting the protomers’ N-lobes and the αC helix dimer interface with the αC helix.

## Introduction

**Figure 1:**
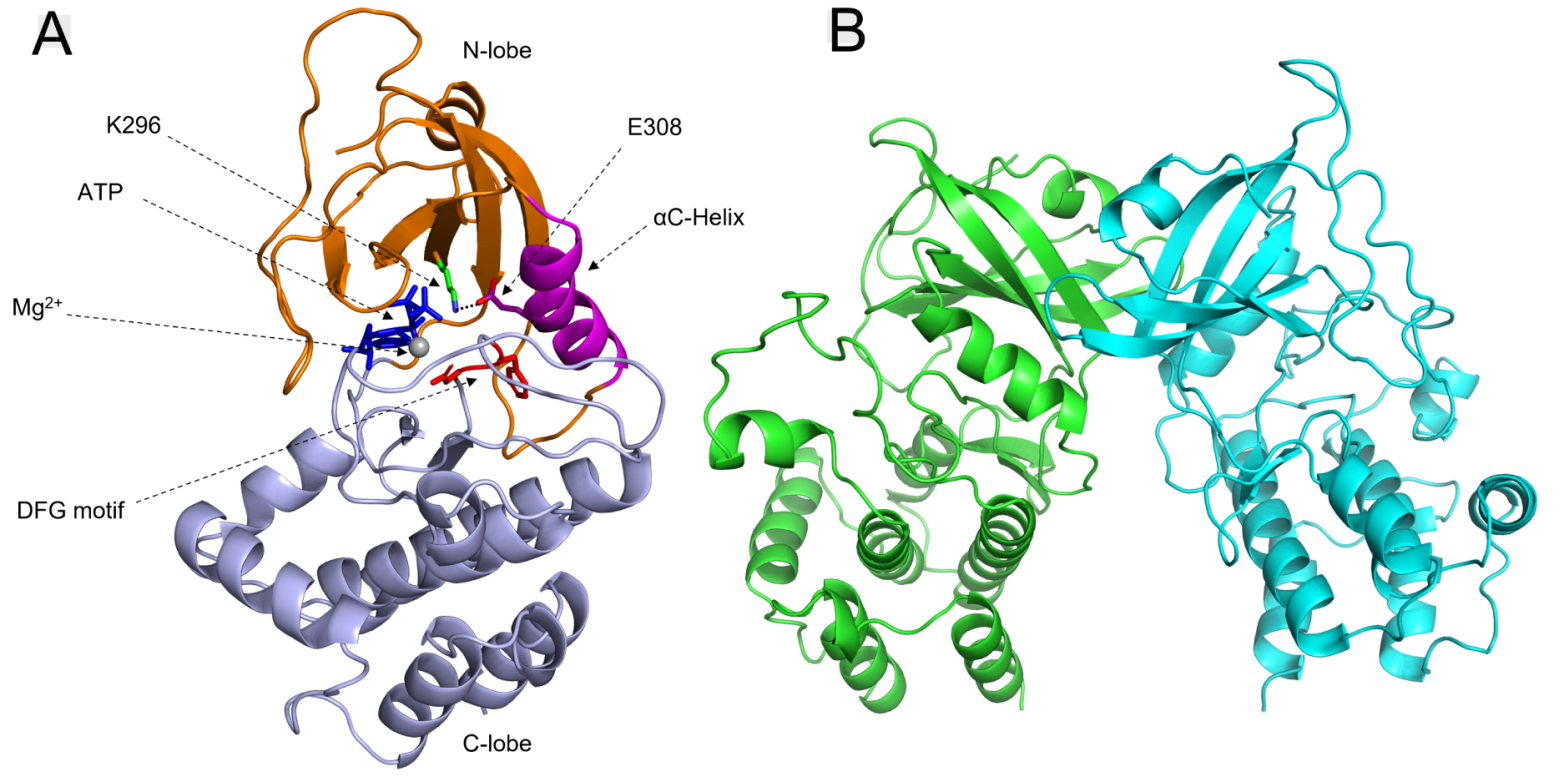
Structure of the PKR kinase domain. (A) The active site lies between the N-lobe (orange) and C-lobe (ice blue). The αC helix is shown in purple. In the active state, E308 of αCH forms a salt bridge with K296, which coordinates the α and β phosphates of ATP. D432 of the DFG motif coordinates with a Mg^2+^ ion. (B) Structure of the back-to-back PKR kinase dimer, where the protomers are colored green and cyan (PDBID: 63KL).

Human protein kinase R (PKR) belongs to the eIF2α family of protein kinases that inhibit translation initiation in response to different stress stimuli. Alongside a range of other roles in metabolic and apoptotic control, PKR functions centrally in the cellular innate immune response to infection by viruses.^1−3^ PKR is activated by viral dsRNA and contains an N-terminal dsRNA binding domain and a C-terminal catalytic kinase domain (KD). Structural and biophysical analyses underscore a pivotal role for dimerization in PKR activation.^4^ The catalytic domain has the typical bilobal architecture found in protein kinases, consisting of a smaller N-terminal (N-) lobe and a larger C-terminal (C-) lobe.^5^ Upon binding viral dsRNA, the PKR kinase domains dimerize via their N-terminal lobe regions with the active sites facing away from each other in a back-to-back (BTB) geometry (Figure 1B)^4,6^ This dimer configuration is believed to induce a prone-to-autophosphorylate (PTA) conformation.^3,6^ PKR is then activated by autophosphorylation and subsequently phosphorylates the translation initiation factor eIF2α, thereby blocking viral protein synthesis in the cell.

The PKR kinase domain comprises several structural elements that are crucial for enzymatic activity (Figure 1A). The ATP-binding site is positioned between the N- and C-lobes. In the C-lobe, an aspartate located with the DFG motif coordinates a magnesium ion. In many kinases, the DFG motif adopts alternative active (DFG-in) and inactive (DFG-out) conformations. In DFG-in conformations, the DFG aspartate’s side chain faces inward; that is, the side chain is pointed toward the catalytic center of the KD, where it coordinates one or more Mg^2+^ atoms which in turn orient ATP for phosphoryl transfer. Conversely, in DFG-out conformations, the aspartate side chain faces away from the ATP-binding site. Generally, the DFG motif has a ratchet-like action: when the aspartate side chain faces in, the phenylalanine faces out, and vice versa. Another conserved regulatory element of kinases is the αC helix (αCH). In the active state, E308 of αCH forms a salt bridge with K296 on β3, which in turn coordinates the α and β phosphates of bound ATP. Displacement of αCH in active kinases can disrupt this salt bridge. Spanning the N- and C-lobes is the hydrophobic regulatory spine (R-spine),^7^ consisting of residues L312, Y323, and F433 in PKR.^8^ In active kinases, the side chains of these residues tend to be stacked linearly, whereas they are unaligned and often separated in inactive kinases.

The formation of the BTB dimer is a key step in the activation of PKR.^3^ Interfacial residues that form interdimer salt bridge and hydrogen-bonding interactions are highly conserved among eIF2α kinases, and mutations that disrupt these interactions block PKR kinase activity.^9,10^ However, the mechanism by which dimerization induces the adoption of an active conformation remains undefined.

In this study, we employ molecular dynamics (MD) simulations of the PKR kinase domain to compute a free-energy profile and analyze the allosteric mechanism for dimerization-induced activation. From analysis of multiple microsecond-scale simulations, we identify αCH as a promising candidate to be a central regulator of PKR activation status and therefore focus on this segment in enhanced sampling simulations, where we calculate the free-energy profiles for activation for both monomeric and dimeric PKR. We demonstrate that the displacement of αCH away from the catalytic center (and the resultant inactivation) is more probable for monomeric than dimeric PKR. Additionally, we perform linear mutual information analysis (LMI),^11^ where we observe correlated inter- and intramolecular dynamics connecting the dimer interface to the rest of the kinase domains. Finally, we perform contact mapping analysis to structurally analyze the dimer interface and determine how the BTB configuration can stabilize the αCH and promote the catalytic activity.

## Methods

### Structure Modeling and Molecular Dynamics Simulations

We conducted MD simulations (Table 1) of the kinase domain of active-state PKR in dimer and monomer forms using an X-ray crystal structure as our initial state (PDBID: 6D3K, chains B and C). In this structure, two PKR kinase domain monomers form a BTB dimer which induces an active-like conformation in the absence of autophosphorylation. Both chains were used in BTB dimer simulations, while only chain C was used for monomer simulations. Unresolved loop regions (chain B: 255, 334–355, 439–451; chain C: 334–356, 441–444, 449) were modeled using Schrödinger Prime v.1.^12,13^ For clarity, throughout the manuscript, we refer to the dimer as composed of chain A and chain B, where chain A refers to the PDB B chain structure and chain B refers to the PDB C chain structure. The completed structures were energy-minimized using the Desmond package^14^ with the OPLS3^15^ force field and reached convergence after 1460 steps. For simulations of ATP containing complexes, the existing ADP was replaced by ATP using UCSF-Chimera, v.4^16^ and manually adjusted with Schrödinger Maestro v.5.^17^ There are no extant inactive structures of the PKR kinase domain: existing structures either have a phosphorylated activation loop and are thus active or are unphosphorylated but still in an active-like conformation. Unphosphorylated monomeric PKR is generally considered to be inactive, and we generated a homology model (Figure S1), based on an inactive crystal structure of a closely related eIF2α kinase, GCN2. The homology models of the inactive PKR monomer were generated with I-TASSER,^18−20^ using the inactive GCN2 kinase structure (PDBID: 1ZYC)^200^ as a template. Compared to the active-state monomer used here, the modeled inactive PKR monomer KD includes 3 additional N-terminal residues (YTV-) and 12 additional C-terminal residues (-KKSPEKNERHTC).

**Table 1:** Simulation Times and Descriptions

System	Replicate #	# of Windows	Simulation Time	Total Simulation Time	Simulation Type	# Atoms	PDBID; Chain	Quaternary Structure
1 – Active Monomer	1	NA	927 ns	927 ns	Equilibrium	71567	6D3K; C	Monomer (active)
2 – Active Monomer	2	NA	3300 ns	3300 ns	Equilibrium	65907	6D3K; C	Monomer (active)
3 – Inactive Monomer	1	NA	973 ns	973 mns	Equilibrium	65027	Homology Model^a^	Monomer (inactive)
4 – Inactive Monomer	2	NA	1200 ns	12000 ns	Equilibrium	65027	Homology Model^a^	Monomer (inactive)
5 – Inactive Monomer	3	NA	1360 ns	1360 ns	Equilibrium	65027	Homology Model^a^	Monomer (inactive)
6 – Inactive Monomer	4	NA	1670 ns	1670 ns	Equilibrium	65027	Homology Model^a^	Monomer (inactive)
7 – Active Dimer	1	NA	837 ns	837 ns	Equilibrium	105141	6D3K; B,C	Dimer (active)
8 – Dimer WEUS	1	24	129 ns	3.1 μs	WEUS	118266	6D3K; B,C	Dimer
9 – Dimer WEUS	2	24	108 ns	2.6 μs	WEUS	118266	6D3K; B,C	Dimer
10 – Monomer WEUS	1	24	114 ns	2.7 μs	WEUS	65955	6D3K; C	Monomer
11 – Monomer WEUS	2	24	108 ns	2.6 μs	WEUS	65955	6D3K; C	Monomer

a Homology Model generated with I-TASSER based on template PDBID: 1ZYC Total simulation time, all simulations: 21.3 μs.

In total, 11 simulations systems were analyzed for this study (Table 1). All-atom MD simulation inputs were generated using CHARMM-GUI^21,22^ and GROMACS 2019.^23^ Throughout, simulations were solvated with TIP3P waters^24^ and ionized with 150 mM NaCl. GROMACS 2019 with the CHARMM-36m^22^ force field was used for all production simulations. In all cases, a cubic simulation box was used. Energy minimization of the dimeric and monomeric equilibrium systems used a steepest-descent algorithm. All systems underwent 100 ps of NVT equilibration followed by 100 ps of NPT equilibration at 300 K and 1 atm. All production runs of the MD simulations were performed in the NPT ensemble, with the pressure isotropically maintained at 1 atm and the temperature at 300 K. Temperature coupling was controlled with a velocity rescaling stochastic thermostat,^25^ with a 1.0 ps time constant. Pressure coupling used the Parrinello–Rahman scheme,^26^ with a time constant of 5.0 ps. All simulation steps utilized particle mesh Ewald summation for calculating long-range electrostatics with a Fourier spacing of 1.2 Å. For van der Waals and short-range electrostatics, GROMACS’ force-switch shifting function was applied at the cutoff distance of 10 Å to smoothly reduce the interaction potentials to 0 at 12 Å. The production runs of the simulations were performed with a 2 fs time step, with frames saved every 10 ps.

### Geometric Analysis of PKR’s DFG Motif and αCH

In the absence of a solved inactive structure of PKR, we sought to identify a regulatory structural element that was likely to modulate PKR’s activity. Toward this end, we focused on two of the kinase motifs most frequently considered predictive of catalytic activity: the DFG motif and αCH. We developed a program based on the KinaMetrix web-based software.^27,28^ KinaMetrix uses a machine learning algorithm, trained on an extensive annotated set of kinase structures, to classify DFG and αCH conformations as active and inactive by scoring them with geometric descriptors using an active structure of the archetypal kinase, protein kinase A (PKA), as a reference. These descriptors include an extended description of the DFG motif as well as αCH angles and dihedrals. Our program was designed to continuously monitor the change in these metrics over the course of several μs long MD trajectories (based on PKR active structures and inactive homology models), rather than scoring the descriptors of a single structure. We use a similar comparison algorithm to score DFG status and a simplified version of the KinaMetrix αCH analysis which is based on the orientation of the vector pointing from the C-terminal end of the helix to its N-terminal end. To analyze angular deviations of αCH, we computed the normalized dot product between the PKR αCH axial vector and a reference vector from chain B of PDBID: 2A19, a previously solved active dimeric PKR structure. The DFG scoring criteria are based on the ratchet-like character of the conserved kinase DFG motif, whose flipping in and out generally involves coordinated rotation of the aspartate and the phenylalanine. In the DFG-in conformation, the DFG aspartate faces the ATP-binding cleft and the phenylalanine points away from it. This conformation is required for effective ATP docking and proper orientation of its phosphate moieties. The canonical DFG-out conformation is reverse, in which the phenylalanine faces (in fact, blocks) the nucleotide binding pocket and the aspartate faces away. Hence, DFG-in is considered an activating conformation, while DFG-out is inhibitory.

Here, a query kinase’s DFG state is defined by two parameters, *D*1 and *D*2, describing vectors orthogonal to the α carbon (Cα) to gamma carbon (Cγ) vector of the aspartate (*D*1) and phenylalanine (*D*2) side chains (Figure S2). Negative values denote a relative angle greater than 90°; positive, less than 90°. Thus, positive values of D1 indicate an aspartate side chain orientated in the same direction as the 2A19 reference (i.e., facing the catalytic pocket, or DFG-in); positive values of *D*2 indicate a phenylalanine side-chain vector facing away from the catalytic pocket (again, this is characterized as DFG-in). The negative corollaries indicate a DFG-out state. The query molecule’s DFG state is given by two comparison vectors for its aspartate  and phenylalanine  (eqs and 1^2^).
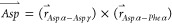
1
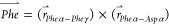
2

where  is defined as the cross product of the vector describing the query aspartate side chain’s angle and the vector pointing from Cα of the DFG aspartate to the phenylalanine Cα.  is similarly given by the cross product of the query Phe side-chain vector and the vector between Asp and Phe Cα. These side chain-orthogonal vectors are used instead of vectors directly representative of side-chain orientation because the orthogonal vectors are insensitive to rotation of the side chains in the plane defined by the DFG backbone and the orientation of the D and F side chains. DFG indicators *D*1 and *D*2 are then scored based on the normalized dot product of the query molecule’s vectors with those of the reference structure, PDBID: 2A19 (eqs and 3^4^):
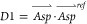
3
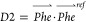
4

*D*1 represents the relative angle between PKR and structure 2A19 aspartate side chain, and *D*2 their phenylalanine side chains.

### Nonequilibrium Molecular Dynamics and Window Exchange Umbrella Sampling

We employed steered molecular dynamics (SMD) and umbrella sampling to compute free-energy profiles of the displacement of αCH from an active(-like) conformation in both monomer and dimer states. In the dimer simulations, chain B αCH is displaced, which is the same chain used in the monomer simulations. Window exchange umbrella sampling (WEUS, i.e., Hamiltonian replica exchange) was performed to improve sampling of PKR’s conformational space along the reaction coordinate by swapping neighboring conformations according to the Metropolis criterion.^29^ To drive the repositioning of the αC helix, SMD was performed with a potential applied between the atoms E308-εO (of αCH) and K296-ζN with a force constant of 2000 kJ mol^–1^ nm^–2^, increasing the distance between the two residues from a starting point of 2.6 Å to approximately 14 Å at a rate of 0.05 Å/ns. This had the effect of displacing αCH away from the catalytic core, emulating a transition from an active state to an inactive state. Simultaneously, a 1,000 kJ mol^–1^ nm^–2^ positional restraint was applied to the backbone atoms of ß-3, in the N-lobe, to prevent structural deformations during the SMD.

Twenty-four frames along this reaction pathway were selected with 0.5 Å spacing to serve as replicas (windows) for WEUS. In each window, the E308-K296 distance was maintained with a harmonic distance restraint (force constant 2092 kJ mol^–1^ nm^–2^). The 24 windows were run concurrently, with an exchange of replicas attempted every 1000 steps (2 ps), based on a version of the Metropolis criterion (eq 5):
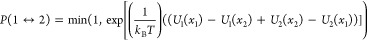
5where *P* is the probability of exchanging two neighboring windows’ coordinates (*x*_*i*_), and *U* is the potential energy, including umbrella restraint, of a replica window. Window exchange probabilities ranged from 0.09 to 0.65, with an average exchange probability of 0.29 (Table S1). Potential of mean force (PMF) curves were computed using the GROMACS version of the weighted histogram analysis method (WHAM).^30^ Two independent SMD and WEUS simulations were run for each system (dimeric and monomeric), and the lowest-energy PMFs calculated for each system were compared. Error estimation was performed by block averaging of the WEUS data, where we computed three PMFs, each using 20 ns of data. PMF errors were computed by taking the standard error of the three 20 ns blocks for each system (Figures S3 and S4).

### Linear Mutual Information Analysis

We used the Bio3D R library^11^ to compute linear mutual information (LMI) matrices of the dynamics in Cartesian space of backbone atoms in dimeric and monomeric equilibrium MD trajectories. LMI was computed on replicate #1 simulations for active and inactive monomers and the dimer simulations (systems #1, 3, and 7 in Table 1, respectively). LMI (unlike Pearson correlation methods, which measure linear relationships) considers both linearly related and orthogonal motions in its correlation calculations, returning a value of 0 (no correlation) to 1 (perfect pairwise correlation in time). LMI figures were plotted with the Matplotlib python library. Pairwise data from LMI matrices produced with Bio3D^31^ were used for the selection of putative allosteric pathways for further analysis. Contact mapping was performed using the GROMACS mdmat tool.^32^

### OHM Allosteric Analysis

To complement the allosteric pathways identified in our LMI analysis, we used the OHM web server.^33,34^ OHM applies percolation theory to determine likely allosteric paths based on interatomic interactions. For our analysis, we used the Identify Allosteric Pathways and Critical Residues tool, which returns ordered lists of residues that are likely to participate in an allosteric pathway connecting selected start and end points. We selected residues L312, A13, and K314 at the C-terminal end of the αCH in chain A as starting points, targeting their connectivity to Y300 of the β4-sheet in chain B, which interacts with chain B’s own αCH.

## Results and Discussion

### DFG-In Conformation Is Dominant in Both Active and Inactive PKR Monomers

Inactive or dysregulated protein kinases can adopt a wide array of conformations. This can make the identification of a central structural regulatory element (and identification of a collective variable (CV) for biased MD simulations) that reliably distinguishes between the active and inactive conformations of the enzyme challenging. Several structural and dynamic features have been identified to characterize inactivation of kinases, including the disruption of the R-spine,^35^ adoption of the DFG-out conformation of the DFG motif,^36^ and displacement of αCH.^37^

**Figure 2:**
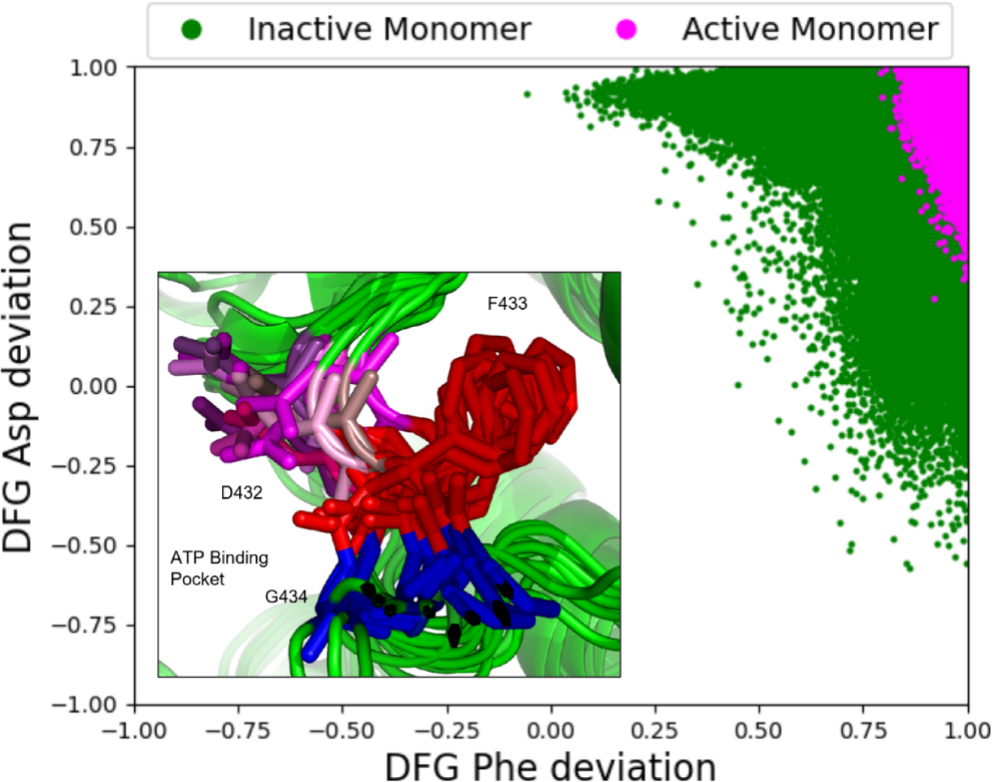
Geometric analysis of the DFG motif. Each data point represents a frame (every 10 ps) from an MD simulation. Indicators are plotted in magenta for the active kinase and green for the inactive kinase. Negative values indicate conformations with the aspartate facing away from the catalytic center or the phenylalanine facing inward, corresponding to inactive states. Inset: ten superimposed structures from an ∼1 μs equilibrium MD simulation of inactive PKR kinase (replicate 1, see Table 1). D432 adopts a variety of conformations but does not undergo the characteristic flip corresponding to the canonical DFG-out state. F433 remains outside the nucleotide binding site.

Geometric analyses^27,28^ were performed on equilibrium MD simulations in which the orientations of PKR’s DFG aspartate (D432) and phenylalanine (F433) were compared to those of an active conformation of a reference PKR structure (PDBID: 2A19). In these analyses (Figures 2 and S5), the dot product of the PKR DFG side-chain vectors with the reference 2A19 vectors was calculated; thus, (1,1) would indicate the DFG-in (active) conformation, whereas a value of (−1,–1) would indicate the DFG-out state (inactive). These analyses indicated low variability of PKR’s DFG motif, and both inactive and active PKR primarily exhibit DFG-in (active) conformations, though D432 demonstrates rare excursions into the negative regime. However, close examination of D432 dynamics shows that it is rarely, if ever, oriented such that its side-chain carboxyl would be unable to interact with Mg^2+^, which coordinates ATP. Furthermore, we never observed any conformations in which both Asp and Phe residues obtain negative values (e.g., lower left quadrant of Figure 2) and therefore never observe a characteristic flip to a DFG-out state. These noncanonical, intermediate DFG conformations would be unlikely to affect the activation state of the enzyme. F433 has even less mobility than D432, especially in the active monomer. F433 is the main steric inhibitor of nucleotide binding in DFG-regulated kinases. As such, it is often called the “gatekeeper” residue,^36^ due to its bulky side-chain’s ability to effectively block the docking of ATP to the catalytic site. The lack of variability in DFG states between active and inactive conformations of the KD in our MD data indicates that DFG status may be a poor predictive metric of PKR activation. Indeed, fewer kinases than previously believed reliably adopt the inactive, DFG-out conformation, calling the binary nature often ascribed to DFG conformation into question.^27,36^ The current KinaMetrix database^27^ contains over 3000 kinase structures where their activation status is characterized based on αCH and DFG conformations. This data set supports αCH as more likely to dictate activation as there are almost three times more structures with αCH-out/DFG-in (665 structures) than αCH-in/DFG-out (232 structures) conformations. However, the time scale of kinase DFG flip (∼ms)^38^ is likely beyond the time scale probed in our simulations, and therefore, we cannot definitively predict that inactive PKR would retain a DFG-in conformation.

### The αC Helix Is a Dynamic Structural Feature and Likely Predictor of PKR Activation Status

**Figure 3:**
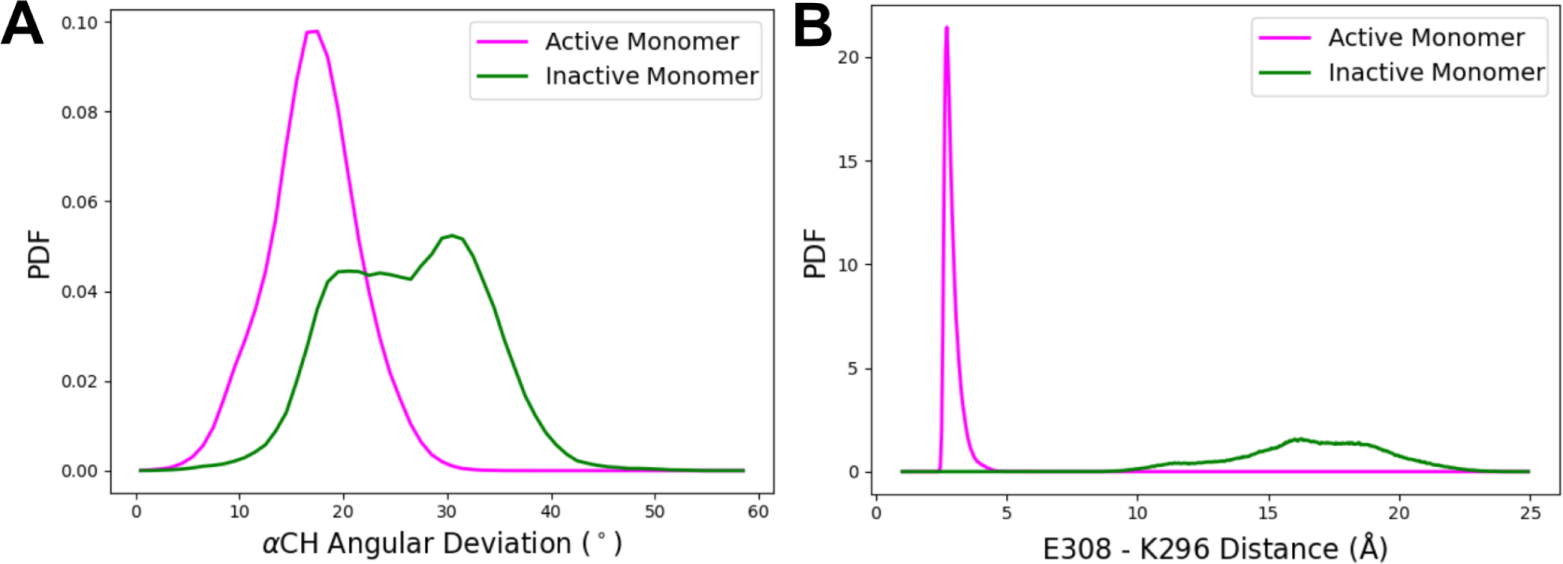
Geometric analysis of the conformation of the αC helix. (A) Probability density function (PDF) of the angular deviation of the αCH from an active reference state. (B) PDF of the E308-K296 bridge distance. PDFs are generated by accumulating all equilibrium replicate simulation data (Table 1).

In contrast to the DFG motif, a geometric analysis of the conformation of αCH showed a significant amount of variability in the inactive state simulation. By measuring the αCH axial vector’s deviation from the 2A19 reference, we clearly observe its greater flexibility in our inactive model (Figure 3A). In equilibrium simulations of the inactive PKR model, the orientation of αCH is quite dynamic. The active state αCH starts with an approximately 5° offset from the 2A19 αC orientation and samples conformations which deviate by almost 30° from that orientation. The inactive helix, however, samples conformations that deviate from 2A19’s orientation by as much as 40°.

αCH also displayed a significant translation away from the catalytic core in the inactive monomer simulations. In contrast, αCH sampled a narrow distribution in the active monomer in which the E308-K296 salt bridge was intact; however, transient excursions to distances over 5 Å and up to 8 Å were observed (Figure S6). We also examined the salt bridge distance distribution in our equilibrium dimer simulation. When the salt bridge distances between the active monomer and dimer are compared, the probability distributions are nearly identical (data not shown). However, the propensity for transient excursions to distances beyond 4 Å is almost twice as likely in the monomer (1.28% of frames) than the dimer (0.77% of frames), indicating a stabilization of the salt bridge (i.e., active state) due to dimerization. On the simulation time scale, no interconversion between active and inactive kinase states was observed. However, the interatomic distance between E308 O to K296 N in the inactive simulations varied by over 10 Å, from 10 Å to over 20 Å (Figure 3B). The demonstrable dynamic flexibility of αCH and its ability to moderate PKR activity by controlling this salt bridge make a strong case for its role as an essential regulatory feature of the kinase.^7,13,37,39 −41^ The αCH motif is universally conserved in Ser/Thr kinases,^37^ and there is strong evidence that it frequently serves as a signal integration hub in protein kinases.^39,41^ Residues from αCH interact with multiple catalytically important kinase motifs,^35^ including the R-spine and elements of the activation loop. In PKR, the E308-K296 salt bridge is sensitive to the conformation and orientation of αCH, as E308 itself is positioned centrally along the helix.^8^ For a more generalized approach, we also computed the atomic root-mean-squared-fluctuations (RMSF) of both DFG and αCH following a local alignment (Figure S7). The results support that αCH is more dynamic than DFG and that both motifs display elevated dynamics in the inactive state. Hence, we chose αCH as the primary focus of our investigation to understand structural determinants and energetics of PKR activation.

### Dimerization Stabilizes the Active Conformation of αCH

**Figure 4:**
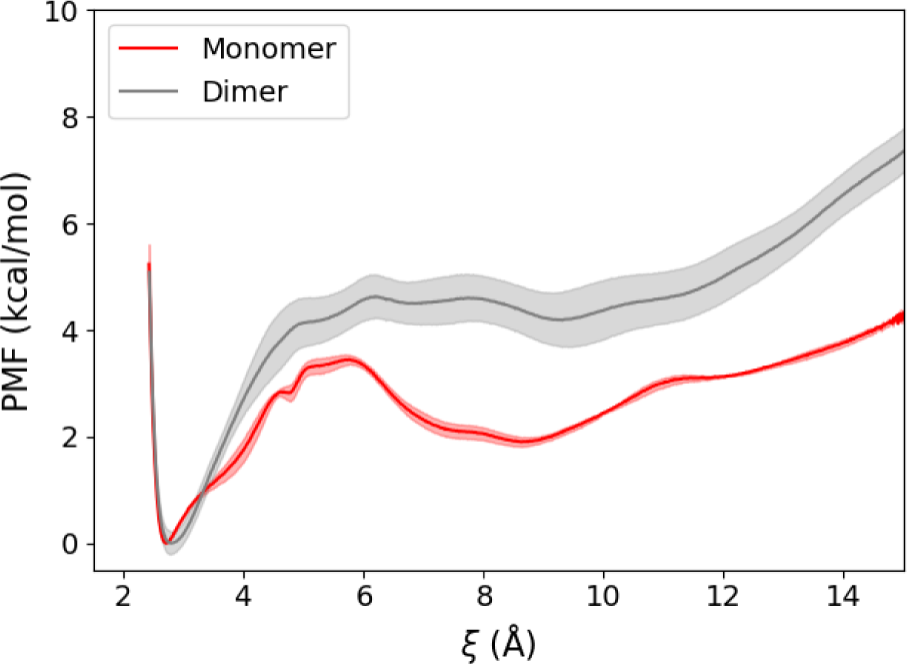
Potential of the mean force (PMF) for αCH displacement. The dimer is in gray, and the monomer is in red. The reaction coordinate (ξ) corresponds to the E308 εO-K296 ζN distance. The most favorable PMFs among the two trials for both monomer and dimer are shown. The shaded area represents the standard error of the mean computed by block averaging in 20 ns increments.

To probe the energetic coupling of dimerization with formation of an active kinase conformation, we employed steered MD simulations of monomeric and dimeric PKR to generate active-to-inactive transition pathways. In these simulations, a force was applied to αCH to cause it to translate, disrupting the E308-K296 salt bridge. WEUS was performed on the transition pathways to obtain the potential of mean force (PMF) for the active to inactive transitions of monomeric and dimeric PKR (Figures 4 and S4). For both monomer and dimer systems, two independent SMD/WEUS trials were preformed (Figure S3). Block averaging shows that each of the dimer trials is well-converged (Figure S3A,B), though there are energetic differences between the trials (Figure S4A). The dimer trials display larger errors than the monomer systems (Figure S3C,D) and again display variability across the trials (Figure S4B). Energetic differences between trials likely arise from generation of independent pathways and the presence of orthogonal energy barriers. We have therefore focused our presentation on the most energetically favorable pathways, as these are the most probable pathways among those sampled. In the lowest-energy pathways, we observe that extension of the E303-K296 distance by ∼8 Å requires approximately 5 kcal/mol for the dimer but less than 3 kcal/mol for the monomer, indicating that dimerization significantly stabilizes the active kinase conformation. Moreover, there is a prominent local minimum at ∼8 Å in the monomer, which is not present in the dimer, resulting in an energy barrier of ∼1 kcal/mol to re-establish the salt bridge in the inactive to active transition. The presence of the barrier in the monomer state indicates that the inactive conformation may be more stabilized compared with the dimer state, where the energy landscape of activation is primarily downhill. We therefore conclude that stabilization of a conformation of αCH that facilitates formation of the critical E303-K296 salt bridge represents a primary mechanism by which dimerization promotes activation of PKR.

### Dimerization Affects Protein Dynamics and Correlated Motions

**Figure 5:**
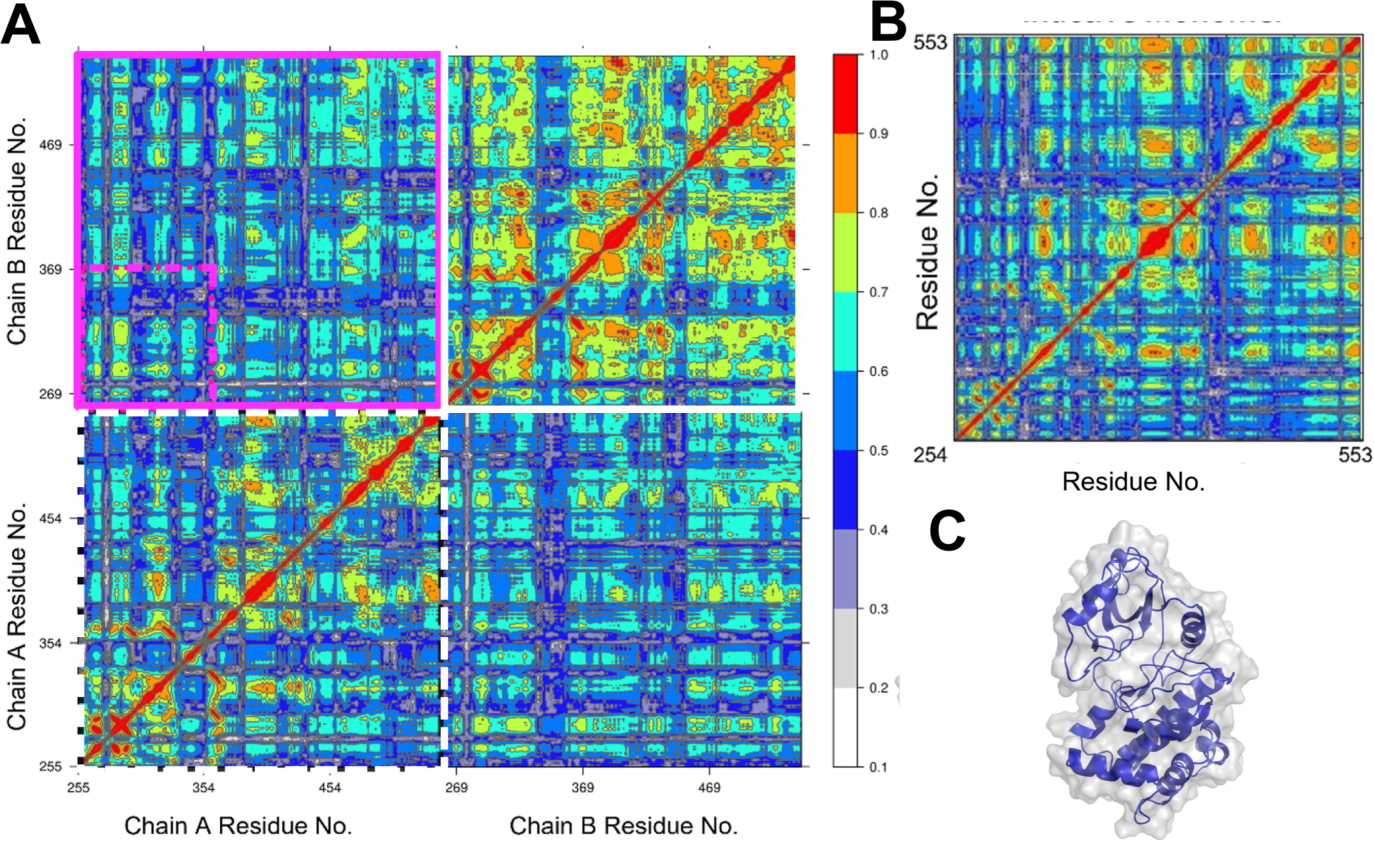
Linear mutual information analysis of PKR kinase. (A) LMI matrix of the PKR dimer. The dashed white boxed region represents dimer chain A, and the solid white boxed region represents dimer chain B. The colors indicate the degree of correlation, ranging from white (no correlation) to red (maximal correlation). The magenta box shows correlations between chains A and B. The small dashed magenta box demarcates correlations between the N-lobes of chains A and B. (B) LMI matrix of an inactive PKR monomer. (C) Structure of the inactive monomer.

Given that dimerization affects the energetics of αCH displacement (Figure 4), which has strong implications on PKR activation, we next performed correlation analysis using linear mutual information (LMI) and contact mapping, to compare dimer and monomer states. Figure 5A shows the temporally correlated dynamics between residues within the PKR BTB dimer. This analysis captures both inter- and intraprotomer correlations, where a value of 0 corresponds to no temporal correlation and 1 means that the two residues are either fully correlated or fully anticorrelated in time. The upper left and lower right quadrants of the matrix represent interprotomer correlations, while the upper right and lower left quadrants represent internal protomer correlations in chain B and chain A, respectively. Interestingly, LMI mapping shows an asymmetric pattern in the degree of intraprotomer correlation, with chain B (Figure 5A, upper right quadrant) displaying considerably more coordinated dynamics than chain A (Figure 5A, lower left quadrant). This may suggest that upon dimerization, the monomer that has the more rigid or coherent conformation is further stabilized. Alternatively, there may be an enzyme–substrate relationship between the protomers in which a given monomer has adopted a conformation capable of preferentially stabilizing its partner upon binding, biasing that monomer toward autophosphorylation. Comparing the correlations in the B chain of the dimer (Figure 5A, upper right quadrant) to an inactive monomer simulation (Figure 5B,C), we observe that the chain participating in the dimer has a distinct and generalized increase in intramolecular dynamic correlation of residues, the inactive monomer shows an average LMI of 0.44, while chain B of the dimer’s average LMI is 0.59. Possibly, dimerization has an overall coordinating effect on the interfacing PKR protomers that accounts for this increase. Close residue packing at the dimer interface produces a greater degree of concerted dynamics, and this correlated motion propagates through the protomer. This is further evident when one chain is removed from its dimeric context (Figure S8A). In terms of LMI, isolated, active-like chain B shows a marked lack of intramolecular correlation after dissociation from chain A, emphasizing the role of dimerization in the synchronization of PKR’s dynamics. Presumably, given enough time under equilibrium conditions, isolated active-like chain B would relax into a conformation conducive to more coordinated motion; the LMI of an inactive dimer under similar conditions (Figure S8B) displays considerably higher correlations than active-like chain B in isolation. This is likely reflective of the fact that the conformation of the inactive monomer (in fact, any monomeric PKR KD that has remained isolated for a sufficiently long time) has equilibrated to an energetically favorable, if enzymatically inert, state.

**Figure 6:**
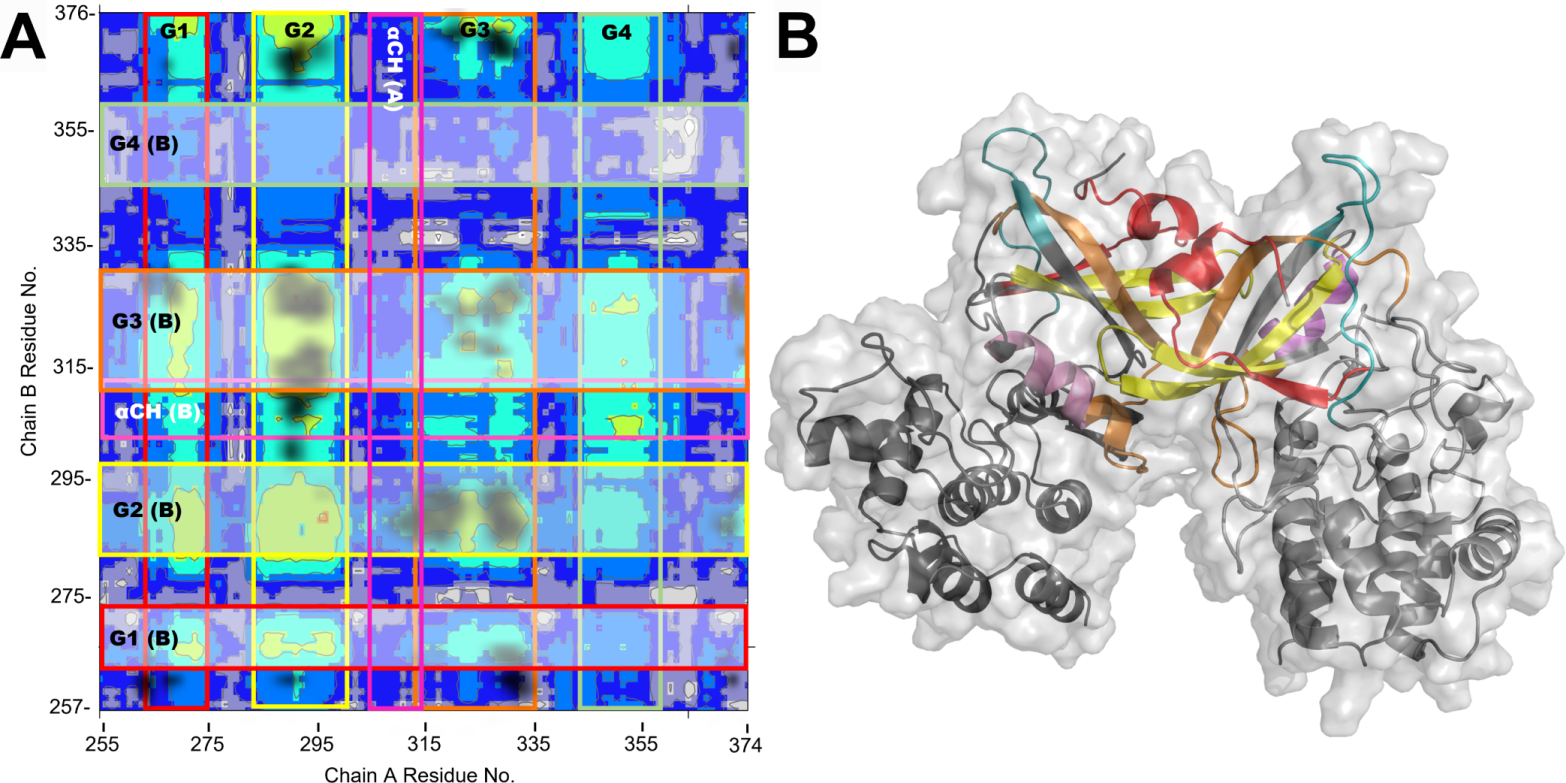
Interprotomer dynamic correlation and contact map between PKR kinase domain N-lobes. (A) A close-up view of the dimer N-lobe interprotomer correlations (dotted magenta box in Figure 5A). Groups of residues in chains A and B that exhibit high correlation are shaded (horizontally, chain A) and/or outlined (vertically, chain B) in red, yellow, orange, and teal. αCH residues are outlined in magenta. Shadowed regions show areas of frequent direct contact. (B) Dimer structure colored by the correlation group. Chain A is shown on the left, and B on the right. Colored structural regions correspond to the groups of high interprotomer dynamic correlation shown in (A). Red: group 1, residues T258-S275; Yellow: group 2, residues F282-Y300; Orange: group 3, residues L312-E335; Teal: group 4, residues D345-K360; Magenta: αCH.

Examining the LMI of the N-lobe region of the dimer in more detail (Figure 6A), four strips or groups of residues exhibiting strong interprotomer correlation (LMI values between ∼0.6 and ∼0.8) are apparent. Notably, these groups include several of the beta strands that comprise the majority of the N-lobe’s more rigid structural features: Group 1 contains ß1, group 2 contains ß4, group 3 contains ß2 and ß3, and group 4 contains the N-terminus of ß5. Group 4, which averages <0.5 LMI, shows less interprotomer correlation than groups 1–3. This group, however, consists mainly of disordered loop residues, so the lack of mutual correlation is perhaps unsurprising.

Residues comprising or adjacent to the interface between N-lobes have a high degree of mutual correlation as well. Frequent contacts between interfacial residues with their conjugate protomer can be seen on the correlation map in the group 2 and group 3 regions, particularly where the A and B chains’ ß3 (R287-K299) and ß4 (N324–Y332) strands intersect, so high dynamic correlation at these sites is to be expected. ß1 (E271-G276), ß3, and ß4 all exhibit high interprotomer mutual information as well. In chain A, these beta strands also show strong correlation with chain B’s αCH. Chain A’s ß3, notably, shows the strongest correlation with chain B’s αCH. This is consistent with the fact that several residues on the amphipathic C-terminal end of the helix interact directly with hydrophobic chain A ß3 and ß2-ß3 turn residues: I288 and Y293. Indeed, it is possible that the close packing between hydrophobic residues of αCH and interfacial residues on the conjugate protomer is contributing to stabilizing the active conformation of αCH in the dimer. Although only a small number of residues make up this region of the interface, the hydrophobic effect could account for the ∼2 kcal mol^–1^ difference observed in the free-energy profiles of the monomer and dimer.^43^

Of particular interest are the areas of the N-lobe LMI map (Figure 6A) that exhibit high correlation in the absence of direct contact. While it might be unsurprising that cognate structures that exhibit frequent explicit residue contacts would be highly dynamically correlated, this correlation appears to propagate from C-terminal αCH residues at the interface (shown in orange in Figure 6A,B) through ß4 and into ß2 and ß3 of both protomers, where no direct contact can be seen. Also notable, the long (Y332 through K358), disordered kinase insert domain (KID) of chain A displays relatively strong correlation with chain B’s αCH as well. While no clear mechanistic role for the KID has been described thus far, there is some evidence that its deletion attenuates or abrogates kinase activity in PKR.^44^

Interprotomer correlation between cognate C-lobes (visible in Figure 5A, as the upper right quadrant of the magenta boxed region) is high. In this region, residues rarely touch, but αF and αG (the binding site for PKR’s primary substrate, eIF2α) of chain A correlate strongly with several chain B N-lobe structures: its αO helix (residues Y261 through D266), ß3, ß4, and ß5. C-lobe to C-lobe correlations exist as well: the αF helices of both protomers share >0.7 LMI; αF, αH, and αJ of chain B, all show strong correlation with chain A’s αF helix. Slow, coordinated “clamshell” or “breathing” motions, in which the N- and C-lobes close around the KD active site, are frequently cited as a hallmark of enzymatic activity in kinases.^45^ The high correlation between the C-lobes may indicate that this motion is being captured by our analysis of the PKR dimer.

These pairwise atomic correlations point to highly synchronized residue dynamics within and between the dimer protomers. The correlations may also suggest a dynamic communication pathway, coordinated by the N-lobes’ beta strands, which connects the protomers’ interface to more distal regions of the KD, such as the C-lobe helices. Concerted, large scale relative motions of the protomers may drive the coherent transmission of residue displacements linearly along the ß strands, maintaining or minimizing the distance between interfacing residues and stabilizing the protomers’ active, more rigid conformation. Furthermore, K296 is located in ß3, which, as mentioned, has a high mutual interprotomer correlation. It is possible that, upon dimerization, the increase in outward-oriented correlated residue motion propagating from the interface and αCH through ß4 and into ß3 contributes to the stabilization of the critical E308-K296 salt bridge in chain B by synchronizing local dynamics. This effect appears to be asymmetric, with a mean LMI above 0.6 for chain B’s αCH with chain A’s ß1 and ß3, but relatively low values for the reverse case, again, dimerization may preferentially affect the stabilization of one protomers’ αC helix or the other.^37,39,41^

To complement our LMI correlation findings, we employed an additional approach (OHM) to identify allosteric pathways between the dimer interface and the active site of PKR. The OHM analysis revealed a highly scored allosteric path (Figure S9), connecting residues at the C-terminal end of the αCH in chain A to β4 of chain B. Notably, this path includes residues L312, A313, and K314, aligning with our selected start points. The correlation data from the OHM analysis recapitulate the interprotomer LMI correlation observed in our dimer model, reinforcing the validity of our proposed allosteric pathways. Specifically, β4 residues K285 and D289-Y293 in chain B scored highly with the αCH of the other chain, consistent with LMI results (Figures 5 and 6).

Our findings on PKR activation align with the dynamic community model of kinase regulation proposed by Taylor and colleagues. In their study on protein kinase A (PKA), McClendon et al. employed community analysis to reveal the dynamic architecture of the kinase.^41^ Their work highlighted the significance of various kinase regions, including the αC helix and the DFG motif, which are organized into functional communities that collectively contribute to kinase activation and regulation. Specifically, in Taylor’s work, the αC helix is part of a dynamic community that acts as a hub connected to six other communities and functions as a major allosteric regulator of activation. Similarly, our results show that the αC helix in PKR interacts dynamically with other elements within the N-lobe and the ATP-binding cleft, which is crucial for stabilization of the active conformation through dimerization. While Taylor’s work discusses the dynamic behavior of the DFG motif as a key element in kinase regulation, our study finds that the DFG motif in PKR remains invariant. This invariance contrasts with the dynamic switching observed in PKA but underscores the specific regulatory mechanism in PKR, where the αC helix plays a more dominant role in its activation.

## Conclusions

Geometric analyses of multiple microsecond-scale MD simulations of active PKR and an inactive PKR model demonstrated that the kinase does not adopt the canonical DFG-out conformation, indicating that this element is not a central regulator of activation (Figure 2). In contrast, αCH shows higher levels of conformational flexibility in the inactive model than in the active state (Figure 3). Along with this, its roles in phosphoryl transfer chemistry and R-spine assembly make it a compelling candidate for PKR’s regulatory hub.

Using enhanced sampling methods, we calculated the free energy of displacement of αCH in a PKR dimer and monomer. The resultant PMFs revealed a greater free-energy difference between the active and inactive states for the dimer, demonstrating that dimerization stabilizes the active conformation of PKR (Figure 4).

Correlation analyses suggest that stabilization and coordination of residues and motifs within the protomers of a PKR dimer may occur asynchronously after they interface rather than simultaneously. Contact mapping points to hydrophobic interactions between αCH and the dimer interface as a possible explanation for the differential free energy for αCH displacement in the monomeric and dimeric configurations of PKR. These findings also suggest that several of the N-lobe beta strands may serve as allosteric conduits connecting the αC helix to distal residues and that allosteric communication along this structure is markedly increased once PKR has dimerized (Figures 5 and 6).

Taken together, our results establish an explicit mechanistic link between dimerization and activation mediated by αCH. As a future experimental approach, the beta strands implicated in communication between the BTB dimer interface and αCH could be subjected to mutational scanning and accompanying activity assays. Future in silico experiments, such as displacing the chain A αCH in the dimer to explore the notion that one protomer acts a “substrate”, could also be merited. Moreover, the suggestion of a dynamic link between the kinase insert domain and αCH might justify a closer examination. This unusually long-loop domain could be more functionally relevant than is currently realized and has been generally neglected in the literature. Here, we have demonstrated that αCH is a pivotal element in PKR activation. While our findings shed new light on kinase regulation, they also underscore that there is terrain still left to explore to fully understand these complex biological systems.

## Supporting Information Available

Figure S1: structural comparison of active and inactive PKR monomers; Figure S2: DFG geometric descriptors; Figure S3: PMFs from 20 ns blocks for all trials and systems; Figure S4: block averaged PMFs for both trials for monomer and dimer; Figure S5: DFG deviation density plots; Figure S6: E308-K296 salt bridge distance in active monomer simulation; Figure S7: DFG and αCH RMSF analyses; Figure S8: LMI comparison between active and inactive monomers; Figure S9: OHM allosteric analysis; Table S1: WEUS exchange statistics (PDF)
