# Supplementary material for: Dimerization Promotes PKR Activation by Modulating Energetics of αC Helix Conversion between Active and Inactive Conformations
Source: J Phys Chem B. 2024 Sep 18;128(39):9305–14. doi: 10.1021/acs.jpcb.4c02460 (PMC11457141; doi:10.1021/acs.jpcb.4c02460)
Supplement: Supplementary file 1 — jp4c02460_si_001.pdf [file jp4c02460_si_001.pdf]

Supporting Information

Dimerization promotes PKR activation by modulating energetics of αC helix conversion between active and inactive conformations

Aaron G. Feinstein,[1] James L. Cole,[1,2] and Eric R. May[1*]

[1]Department of Molecular and Cell Biology, University of Connecticut, Storrs, CT 06269, USA

[2]Department of Chemistry, University of Connecticut, Storrs, CT 06269, USA

[*]Correspondence: eric.may@uconn.edu

Supporting information contains: supplemental Figures S1-S9 and supplemental Table S1.

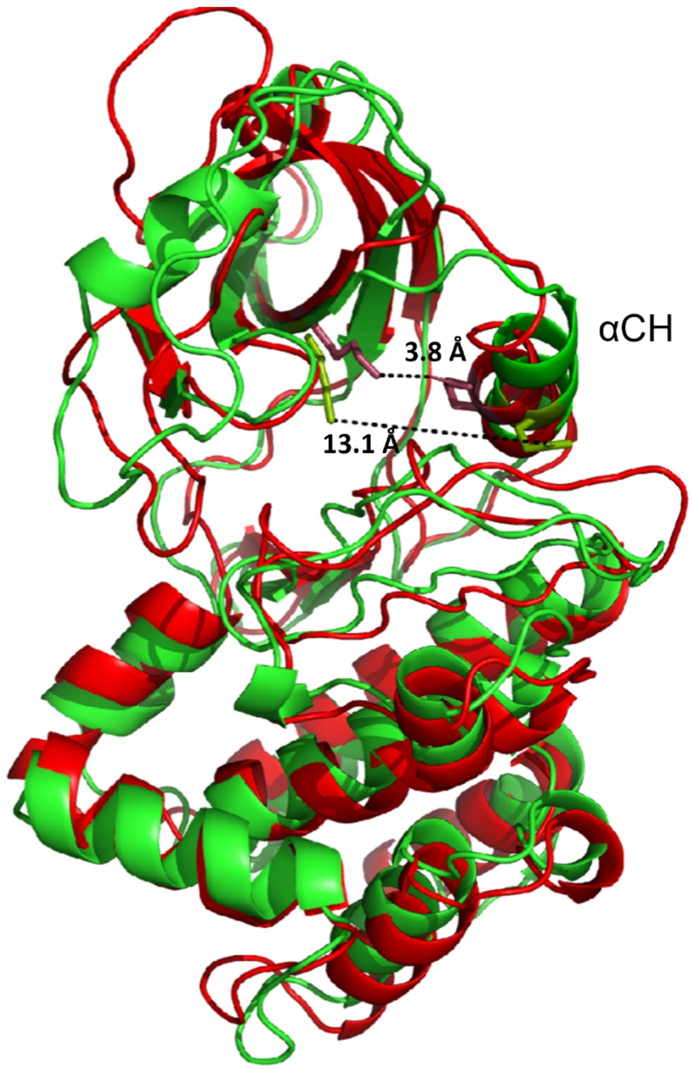

Figure S1. Comparison of the structures of inactive and active PKR kinase. The model of inactive PKR kinase (green) is superimposed on active PKR kinase (PDBID: 6D3K; red). The E308- K296 distances are indicated. Helix *α*CH in the inactive structure is in a conformation with its N-terminal end pointing outward, thus disrupting the E308- K296 salt-bridge.

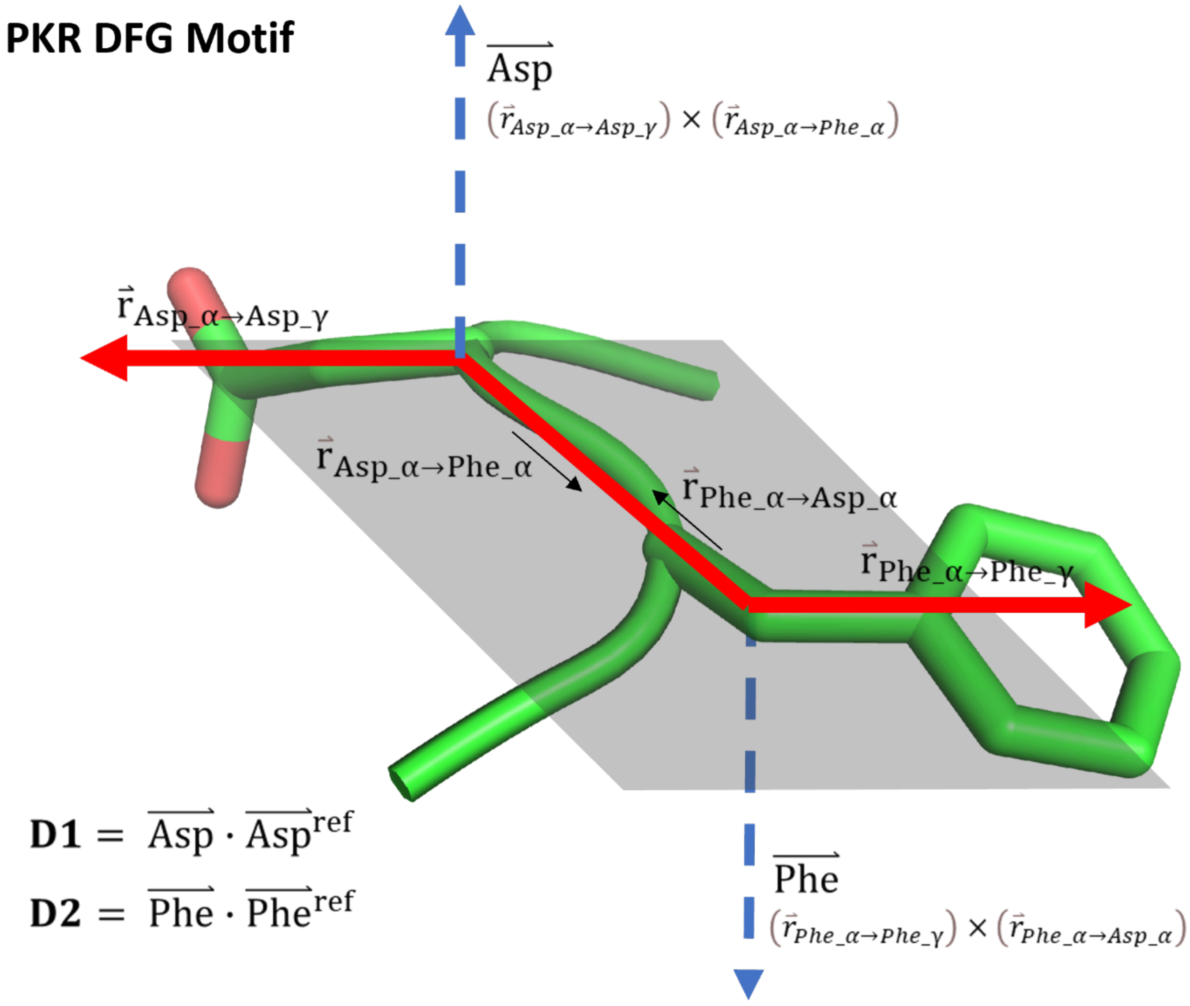

Figure S2. Geometric Analysis of DFG. PKR's DFG motif is scored with descriptors D1 and D2. The dot product between PKR and a PKA reference is calculated between vectors orthogonal to the plane described by the enzymes' Asp and Phe sidechains and the bond between them.

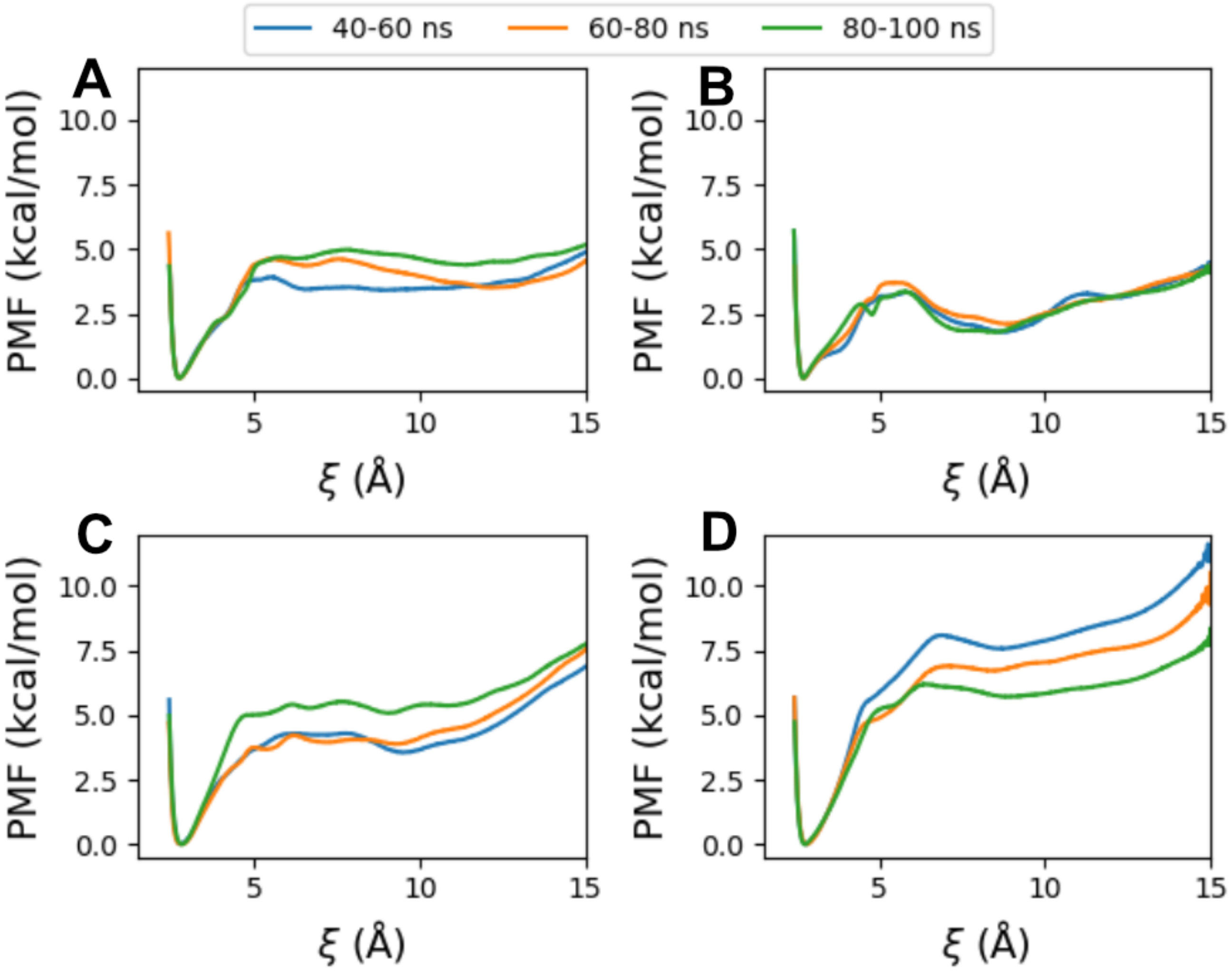

Figure S3. Convergence of PMFs. Block PMFs from WEUS are shown for monomer systems (**A-B**) and dimer systems (**C-D**). Panels **(A), (B), (C)** and **(D)** correspond to systems 11,10, 8 and 9 in **Table 1** (and **Table S1)**, respectively.

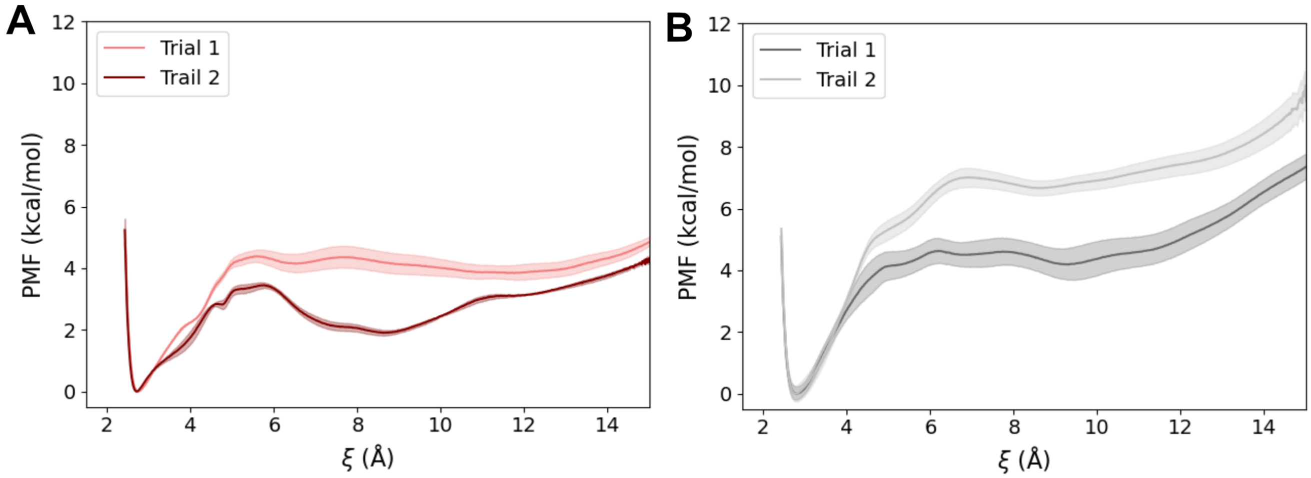

Figure S4. WEUS replicate PMFs. PMFs computed using WEUS from independent SMD trials for a monomeric system **(A)** and a dimeric system **(B)**. Error bars are the standard error computed over the three 20 ns blocks per system (block PMFs shown in **Fig. S3)**.

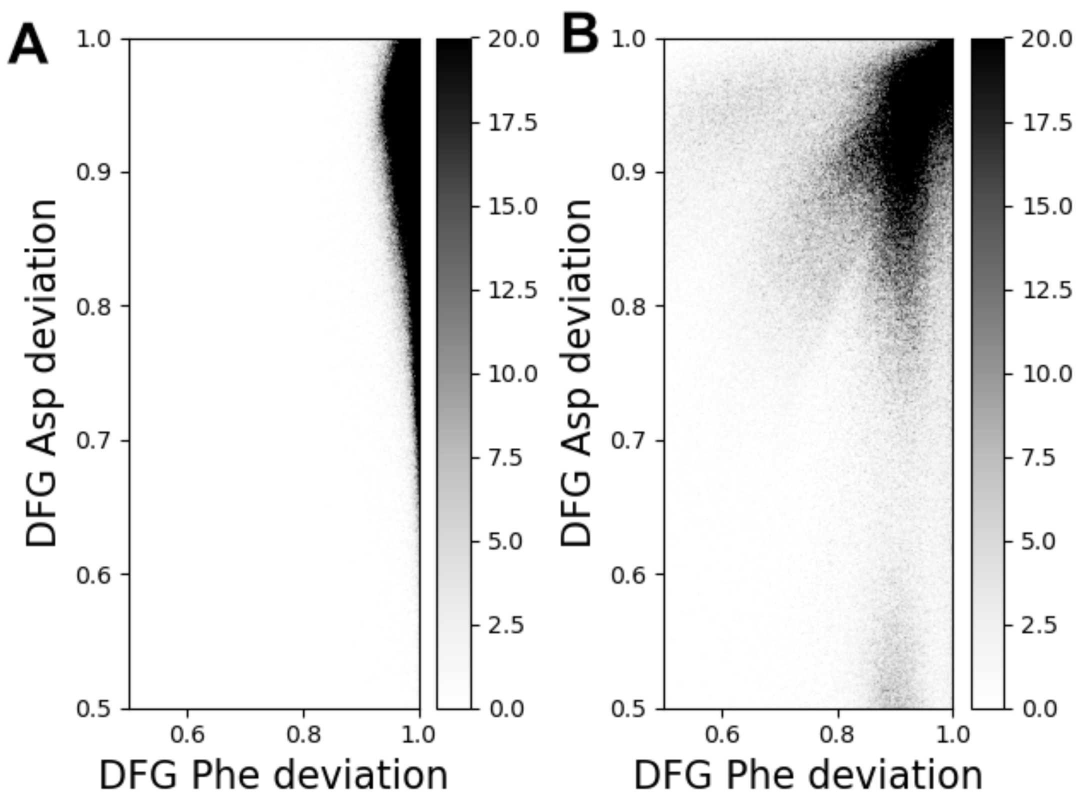

Figure S5. DFG deviation density plots. Density plots for active **(A)** and inactive **(B)** equilibrium simulation data sets are presented. Plots were generated by histogramming data shown in **Fig. 3**. Note that axes ranges are zoomed in to the upper right quadrant to illustrate differences in data sets.

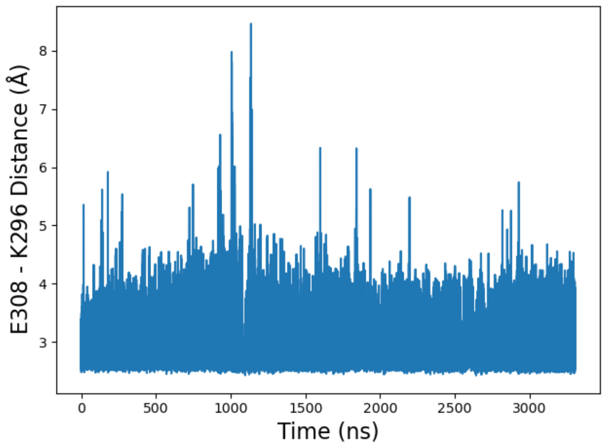

Figure S6. Active monomer E308-K296 salt bridge distance. The salt bridge distance is measured for replicate 2 for the active monomer simulation.

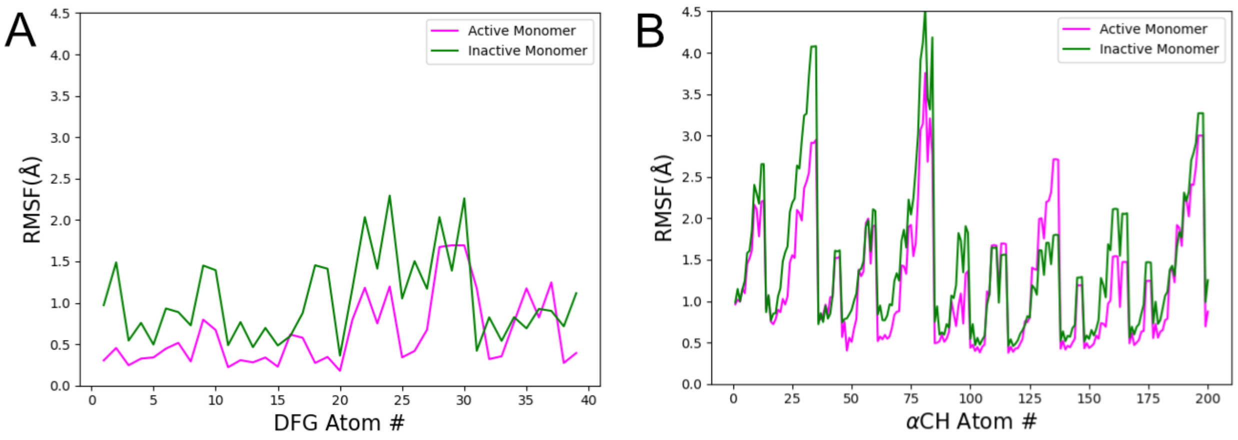

Figure S7. RMSF analysis. RMSF analysis was performed following local structural alignments on DFG **(A)** and αCH **(B)**. In both figures, RMSF curves are averaged over all replicates (see Table 1).

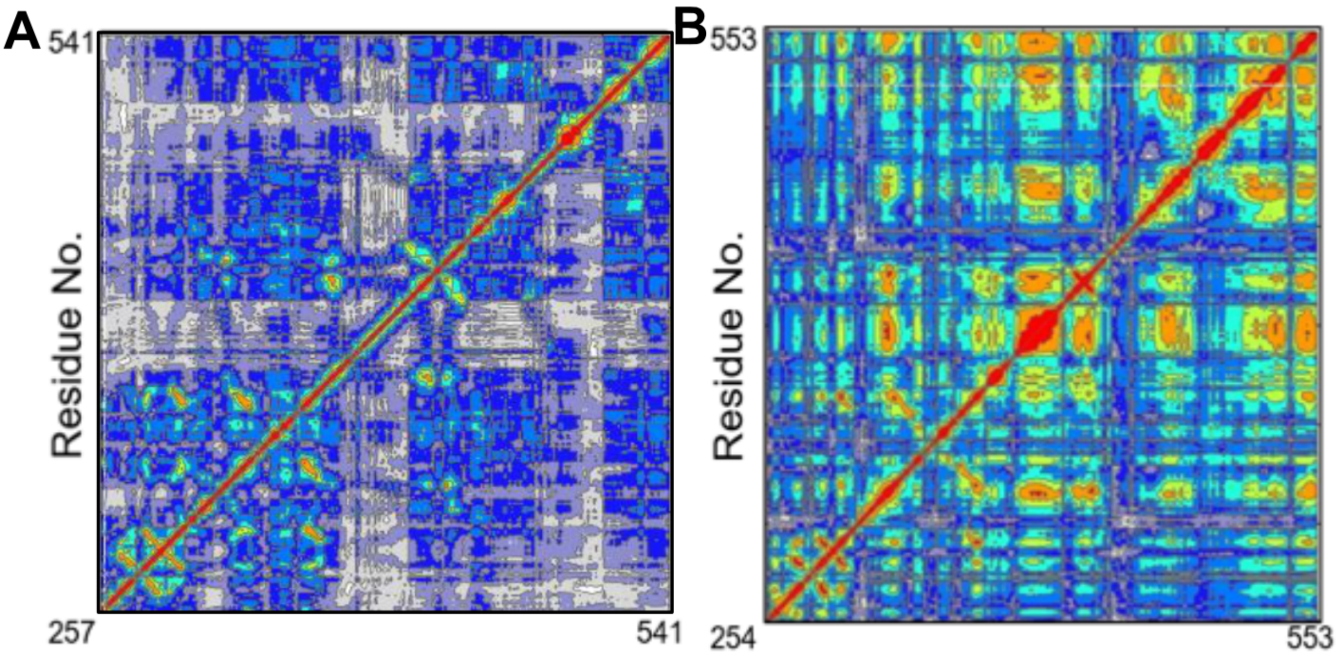

Figure S8. LMI of isolated dimer chain B and inactive monomer. (A) Separating the chain B protomer from its conjugate causes significant generalized decorrelation of its residues (system 1, **Table 1**). **(B)** In the inactive model (system 3, **Table 1**), residues are more correlated than the dissociated chain (**A**), suggesting that PKR KDs removed from their oligomeric state relax into more synchronous dynamics after a period of equilibration, though they do not attain the degree of correlation they display while participating in a homodimer.

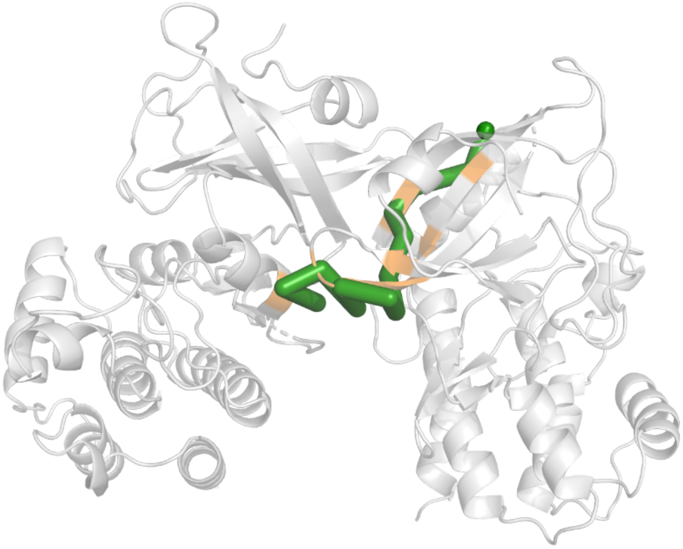

Figure S9. **OHM allosteric analysis.** The highest scoring allosteric path identified by the OHM web application, starting from residues L312, A13, and K314 at the C-terminal end of the αCH in chain A, and connecting to β4 in chain B. The path is highlighted in green, illustrating the dynamic coupling between these residues.

Table S1. WEUS replica exchange statistics. Exchange probabilities for all WEUS systems.

System*	State	Run Time (ns)	HREX Window (Average probability of Hamiltonian exchange between neighboring umbrella windows)																						
			0-1	1-2	2-3	3-4	4-5	5-6	6-7	7-8	8-9	9-10	10-11	11-12	12-13	13-14	14-15	15-16	16-17	17-18	18-19	19-20	20-21	21-22	22-23
8	Dimer**	129	0.43	0.27	0.09	0.18	0.17	0.18	0.17	0.16	0.21	0.20	0.18	0.25	0.17	0.25	0.24	0.27	0.28	0.27	0.27	0.14	0.25	0.25	0.27
9	Dimer	108	0.63	0.50	0.46	0.42	0.19	0.26	0.34	0.27	0.30	0.35	0.36	0.38	0.32	0.30	0.38	0.39	0.36	0.37	0.35	0.36	0.36	0.38	0.39
10	Mono**	114	0.60	0.43	0.34	0.26	0.23	0.27	0.24	0.30	0.35	0.37	0.32	0.31	0.32	0.33	0.31	0.29	0.31	0.30	0.31	0.31	0.32	0.32	0.31
11	Mono	108	0.46	0.23	0.16	0.10	0.13	0.20	0.20	0.17	0.18	0.19	0.19	0.18	0.13	0.17	0.19	0.20	0.20	0.18	0.20	0.21	0.21	0.18	0.13

* For system details, refer to Table 1

**PMFs for these systems are shown in Figure 4
